# Properties and anti-fungal activity of liquid by-products from softwood bark carbonization

**DOI:** 10.1186/s40643-025-00875-8

**Published:** 2025-04-24

**Authors:** Mariem Zouari, Faksawat Poohphajai, Kristine Meile, Marica Mikuljan, Rene Herrera Diaz

**Affiliations:** 1 InnoRenew CoE, Livade 6a, 6310 Izola, Slovenia; 2 Faculty of Mathematics, Natural Sciences, and Information Technologies, University of Primorska, Muzejski Trg 2, 6000 Koper, Slovenia; 3 Andrej Marušič Institute, University of Primorska, Titov Trg 4, 6000 Koper, Slovenia; 4 Department of Bioproducts and Biosystems, Aalto University School of Chemical Engineering, 00076 Aalto, Finland; 5 Biorefinery Laboratory, Latvian State Institute of Wood Chemistry, Str. Dzerbenes 27, Riga, 1006 Latvia; 6 ‘Materials + Technologies’ Group (GMT). Department of Chemical and Environmental Engineering. Faculty of Engineering, Gipuzkoa, University of the Basque Country UPV/EHU, Donostia-San Sebastián, Spain

**Keywords:** Pyrolysis liquid, Valorization, Bark, Antifungal compounds

## Abstract

This study investigated the potential use of pyrolysis liquid from bark as an anti-fungal substance against food decaying fungi. Four different fractions of pyrolysis liquid were collected during variable temperature phases of the pyrolysis process: F1 (25–260 °C), F2 (260–512 °C), F3 (512–800 °C), and F4 (800–25 °C). The thermal degradation of bark material was assessed using TGA analysis. The concentration, pH, total phenolic content, and functional groups of the liquid samples were determined. Additionally, the molecular composition was examined using UHPLC and QToF mass spectrometry methods. Fungal species were isolated from bell pepper and animal fat and identified through microscopic observation and DNA sequencing. The anti-fungal activity of the liquid fractions was evaluated using the disk diffusion test. The obtained degradation thermograms had a typical shape characteristic of lignocellulosic materials, revealing different thermal degradation phases of the bark. These phases served as a basis for the collection of the pyrolysis liquid in fractions, which were expected to differ in properties and molecular composition. In the fractions collected above 260 ºC (F2, F3, F4), the pyrolysis liquid presented an acidic character, resulting from the complex thermochemical reactions that occur during the slow pyrolysis of bark. F2 had the highest concentration of total phenolic compounds (6.46 mg GAE/g extract) while F1 and F4 contained only negligible amounts. The FTIR spectra of F2 displayed additional peaks compared to other samples which provided information on the occurrence of various compounds. The reversed phase UHPLC-UV analysis revealed that furfural, 5-hydroxymethyl furfural, and 5-methyl furfural were the most abundant compounds, and F2 had the highest concentration of summed furans (570 µg/mL) among all samples. The morphological assessment and DNA sequence analysis of the fungal strains revealed that *Penicillium crustosum* and *Cladosporium* sp were isolated from fat and bell pepper, respectively. The antifungal activity of the liquid fractions was limited due to their low concentration (ranging from 0.24% to 0.01% (v/v)), with only minor inhibition observed for F2, indicated by a small inhibition zone of approximately 1 mm around the 10 mm filter paper. However, concentrating the fraction F2 up to 1% (v/v) demonstrated a stronger inhibitory zone against *Cladosporium pseudocladosporioides* and *Penicillium* sp., indicating its antifungal potential at higher concentrations. Overall, the pyrolysis liquid demonstrated promising antifungal activity, particularly after concentration, with F2 exhibiting the highest bioactivity and strongest inhibition effect. These findings highlight its potential for controlling food-decaying fungi while emphasizing the need for further purification, toxicity assessments, and application studies to ensure its feasibility for agro-industrial applications.

**Graphical Abstract:**

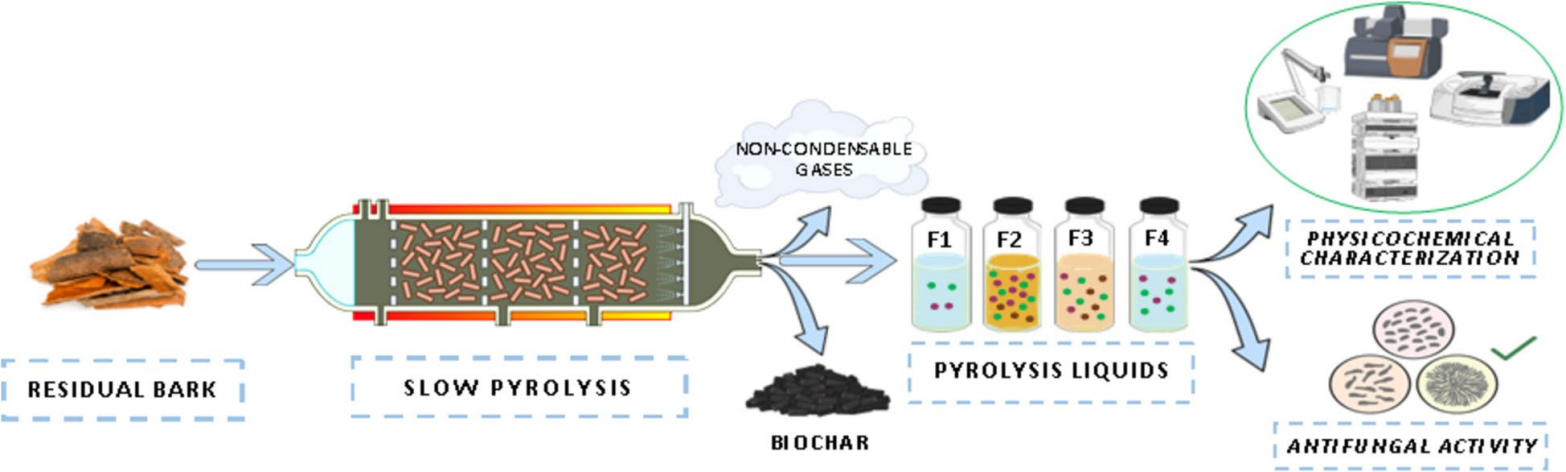

**Supplementary Information:**

The online version contains supplementary material available at 10.1186/s40643-025-00875-8.

## Introduction

The emergent need for managing residual fractions derived from thermochemical processes has stimulated research to valorize side streams and by-products generated by different processes. Thermal conversion technologies have been recognized as an effective route for the conversion of almost any type of under-utilized organic feedstock into high added-value products. One example of these technologies is the slow pyrolysis process, which consists of the degradation of biomass at high temperatures in an oxygen-free atmosphere and a heating range from 0.1 to 0.8 °C/s (Basu 2013). The slow pyrolysis generates three co-products consisting of a major fraction of solid residue called biochar, along with condensable and non-condensable gas fractions (Fagernäs et al. 2012).

Biochar is recognized as a functional material in several applications due to its interesting properties, specifically, large porosity, presence of surface functional groups, and electrical conductivity. For instance, biochar has been utilized as an additive in reinforced-polymeric composites (Zouari et al. 2022), sensors (Spanu et al. 2020), and as an adsorbent for environmental remediation (Xiang et al. 2020; Zouari et al. 2023). Depending on the biomass type and pyrolysis conditions, the process typically yields 15 to 40% of solid biochar (Brown et al. 2011). The remaining volatile fraction consists of condensable and non-condensable gases produced during the slow pyrolysis and are the least valorized products of the process.

The condensable gas fraction is recovered using a condensing system (Papari and Hawboldt 2018) resulting in a non-negligible amount of liquid side stream called pyrolysis liquid. The characterization and identification of bioactive compounds eventually occurring in the pyrolysis liquid represent a promising route for exploring potential applications of the by-product which can upgrade the economic value of the pyrolysis process. In this view, researchers have attempted to investigate the condensable gas fraction derived from the pyrolysis of different types of biomass.

For instance, Velghe et al. (2013) investigated liquid by-products from slow and fast pyrolysis of sludge residue. They found that for both types of pyrolysis, the water-rich fraction contained about 85% of aliphatic compounds such as aliphatic acids and aliphatic amides while aromatics represented 5%. They concluded that the water-rich fraction has a very low heat of combustion value making it unsuitable as a fuel source. In another study (Amini et al. 2019), the yield and composition of volatiles collected using a cold water condenser from pyrolysis of 14 different plant species were studied. It was reported that the pyrolysis liquid yield was up to 55% and that phenolics were the main detected components. Similarly, Marrot et al. (2022) reported the presence of phenols, namely guaiacyl and syringyl, as well as furans in pyrolysis liquids derived from hemp. Additionally, it was found that the phenolic compounds exhibited a remarkable antioxidant activity which increased the potential of these molecules as additives in different applications after adequate extraction and purification.

The above literature focused on the characterization of pyrolysis liquid while only a few studies have proposed potential applications for the identified molecules, mainly for wood protection. For instance, Dellarose Boer et al. (2021) examined the anti-fungal and anti-termite properties of the chemical compounds from sugarcane pyrolysis liquid. The detected compounds consisted mainly of acetic acid, glycolaldehyde, 1-hydroxy-2-propanone, methanol, formic acid, levoglucosan, and furfural, as well as smaller amounts of phenols derivatives. Authors stated that low concentrations of pyrolysis liquid (0.20% and 0.25% v/v) were effective in completely inhibiting the growth of *Coniophora puteana* and *Trametes versicolor* in Petri dishes. Additionally, they reported that 10% of pyrolysis liquid on filter paper caused 100% mortality in termites. Finally, authors concluded that pyrolysis liquid can be potentially used for the preparation of products to protect wood against biological decay. Moreover, a high concentration of total phenols from bamboo and rubberwood pyrolysis liquids acted as highly efficient antifungal agents (Theapparat et al. 2015). Likewise, pyrolysis distillates from bark materials (spruce and birch) and hemp fibers inhibited the growth of wood-decaying fungi (Barbero-López et al. 2019).

In general, most research has focused on pyrolysis liquid as a potential agent for wood protection. However, using pyrolysis liquid as an agent against food spoilage fungi has been less explored. In terms of food, pyrolysis of biomass has been proposed for the production of liquid smoke as a flavouring agent (Xin et al. 2021), and also as a preservative (Faisal et al. 2025). It is important to note that liquid smoke is a food safe product prepared in controlled conditions. Indeed, the liquid smoke is generated by condensation, then it undergoes phase separation, purification, and aging (Xin et al. 2021). However, untreated pyrolysis liquid is chemically unstable and the application of pyrolysis products for human consumption raises food safety concerns.

In summary, several studies have demonstrated the antifungal properties of pyrolysis liquids but they have not focused on food preservation applications and have often overlooked critical limitations such as potential toxicity, chemical instability and rigorous safety assessments. It is worth clarifying that the present study aimed to investigate the potential of compounds present in pyrolysis liquid as food preservatives by evaluating their antifungal efficacy specifically against food-decaying fungi. However, the use of these liquid fractions is not recommended without proper purification and the necessary toxicity and safety tests to meet food safety standards.

In this study, specific actions were implemented to address some technical limitations, for example, in situ fractionation of pyrolysis liquid by temperature ranges to align with the thermal degradation phases of lignocellulosic materials, helping in the chemical identification and controlling the stability of key bioactive compounds, such as phenolics and furans. Furthermore, the obtained soluble and insoluble fractions were filtered through a 2µm-particles size filter, facilitating further concentration of target compounds and thus adjusting the potential use as sustainable food preservatives. However, as these were preliminary screening tests, additional safety considerations are essential before scaling up and applying these findings in the food industry.

This research advances the understanding of pyrolysis liquid by demonstrating its antifungal activity in a food-related context while implementing a targeted fractionation strategy to enhance its stability and bioactivity. These findings provide a basis for further investigations into the controlled application of pyrolysis liquids, linking their chemical composition to their potential use as bio-based food preservation agents.

The objectives of this study were to i) investigate the properties and molecular composition of pyrolysis liquid from bark, and ii) evaluate the anti-fungal potential of pyrolysis liquid against food decaying fungi.

## Materials and methods

### Thermogravimetric analysis of raw bark

Thermal degradation behaviors of raw bark material were characterized by thermogravimetric analysis (TGA) (GA 5500, TA Waters Instruments, New Castle, DE, USA). The bark material consisted of a mixture of spruce and pine bark provided by Holmen (Stockholm, Sweden). Approximately 5 mg of the bark powder was placed in platinum pan, and the degradation thermogram was collected at a temperature range from 25 to 800 °C. Measurements were carried out under N_2_ atmosphere with a flow rate of 25 mL/min and a heating rate of 25 °C/min. The analytical parameters (i.e., temperature and heating rate) were selected to mimic the pyrolysis process in the tube furnace.

### Collection and characterization of the pyrolysis liquid

Pyrolysis liquid fractions were collected during slow pyrolysis of bark material. 200 g of bark were placed in thermo-resistant crucibles. The crucibles were then placed in a tube furnace (Nabertherm RSRC 120–1000/13, Nabertherm, Lilienthal, *Germany*) and pyrolyzed under a N_2_ flow rate of 300 L/h and a heating rate of 1500 °C/h. The pyrolysis time was set to 30 min and temperature was raised from 25 °C to 800 °C and three liquid fractions were collected every 10 min (F1, F2, and F3). A fourth (F4) fraction was collected after the end of the pyrolysis process (i.e., during the cooling down of the tube furnace from 800 °C to ambient temperature). The pyrolysis was conducted in triplicated, and the mass concentration of each fraction was calculated as the mean standard deviation of the three independent runs. Moreover, statistical comparisons between the pyrolysis fractions were performed using OriginPro software (V. 2015), applying a one-way analysis of variance (ANOVA), followed by Tukey's post-hoc test for multiple comparisons. A p-value < 0.05 was considered statistically significant.

The temperature ranges for F1-F4 were selected to automate the collection during the pyrolysis process. The phases were programmed at 10-min intervals, resulting in temperature ranges that correspond approximately to the degradation regions of the main lignocellulosic components: hemicellulose, cellulose, and lignin. These phases were previously verified by the thermogravimetric analysis (TGA) data, identifying the different regions where the material presented weight loss. The temperature ranges of the different fractions, along with the corresponding time intervals and decomposition phases, are summarized in Table 1.

**Table 1:** Collection of different pyrolysis liquid fractions

Sample ID	Temperature range, °C	Phase of collection	Phase of decomposition
F1	25–260	0–10 min of pyrolysis	Initial: low-molecular-weight volatiles such as organic acids and alcohols
F2	260–512	10–20 min of pyrolysis	Main: hemicellulose, cellulose and partly lignin, majority of bioactive compounds
F3	512–800	20–30 min of the pyrolysis	Final: lignin, complex phenolic structures, degraded products
F4	800–25	Post-pyrolysis (cooling down)	Residual: remaining volatiles

Each fraction was filtered in a stainless steel pressure Filter (Sartorius, Germany) using cellulose filters with a pore size of 2 μm. Concentrations of the solid and filtered solutions were calculated, and samples were stored under stable conditions (at ± 4 °C or frozen), prior to further analysis. The mass yield of each fraction was calculated as the mean ± standard deviation (SD) of the three independent runs.

The pH of the different fractions was determined using a pH meter equipped with an IS-68X591206-B-VSTAR pH/LogR Module (Orion Versa-Star Pro Meter, Thermo Fischer Scientific, Waltham, Massachusetts, USA). Three repetitions were performed for each sample and the average values were reported as well as the as the mean standard deviation.

The total phenolic content (TPC) of each fraction was measured following the Folin–Ciocalteu method (Goldberg et al. 1999). First, serial dilutions of gallic acid solution in methanol were prepared and used later to draw a calibration curve. Then, 0.3 mL of the solution to be tested (each fraction and gallic acid solutions) was mixed with 2.5 mL of aqueous Folin–Ciocalteu reagent (10% w/v). The mixtures were then covered for 30 min, and the absorbance of all the solutions was measured at 765 nm with a UV–VIS Spectrophotometer UV7 (Mettler Toledo, Columbus, OH, USA), using methanol as a blank. The calibration curve was obtained by using gallic acid, and the total phenolic compounds of the samples were expressed in milligram gallic acid equivalent per g of dry extract (mg GAE/g). The experiment was repeated in triplicate, and the mean value and standard deviation were reported.

The functional groups were analyzed using a Fourier-transform infrared (FTIR) spectrometer (Alpha FT-IR Spectrometer Bruker, Billerica, MA, USA) connected to an ATR (attenuated total reflection) module. The spectra were collected over a wavelength range from 400 to 4000 cm^−1^, and a resolution of 4 cm^−1^. For each sample, ten replicates were performed with 64 scans per repetition to increase the accuracy of the results and minimize the effect of the atmospheric noise. Opus software was used to collect average spectra, which were further treated by eliminating CO_2_ and atmospheric water vapor effects.

The chemical composition was investigated using ultra-high performance liquid chromatography (UHPLC). A Waters Acquity HSS C18 column (2.1 × 100 mm, 1.8 µm) at 30 °C was used. The gradient with 0.4 mL/min flow rate consisted of water with 0.1% formic acid and acetonitrile (acetonitrile 5% from 0 to 0.5 min, increased to 50% at 6 min, then 95% at 7 min, maintained so until 7.3 min, then returned to initial conditions). Detection was performed with a Waters PDA photodiode array detector at 280 nm wavelength. The samples were filtred through a Kinesis syringe filter (nylon, 0.22 µm). The injection volume was 2 µL. Three main chemical products were identified in the chromatograms and quantified with the calibration curves of the corresponding standards—5-hydroxymethyl furfural, 5-HMF (≥ 99%, Sigma-Aldrich), furfural (99%, Sigma-Aldrich) and 5-methyl furfural, 5-MF (99%, Sigma-Aldrich). The Quantification of the main chemical products was presented as the mean concentration and standard deviation of three replicates.

To perform QToF mass spectrometry, the same UHPLC conditions described above were used in combination with high resolution mass spectrometry (Waters Synapt G2-Si). Untargeted analysis was done in negative ESI mode with trap collision energy 5 V, and cone voltage 40 V. Leucine enkephalin (Waters) 0.2 ng/mL solution in water/acetonitrile (50:50 v/v) was used as lock mass with flow rate of 10 µL/min.

### Antifungal activity test

*Isolation of food-decaying fungi* The fungal strains utilized in this study were isolated from spoiled food, specifically bell peppers and animal fat. In brief, fungal inoculums were obtained using sterile cotton swabs and cultured on Potato Dextrose Agar (PDA) for seven days in the dark within a conditioning chamber set at 25 °C and 90% relative humidity (RH). Following incubation, a representative colony displaying the most prevalent morphology, as determined through visual assessment, was selected from each plate and isolated to obtain a pure culture on PDA plates. The pure cultures were then incubated in the dark under control climate conditions at 25 ℃ in the growth chamber for seven days. The isolated strains were identified based on visual observations of macro morphology (growth patterns, colonies shape, and color) and microscopic observations (EVOS M7000, ThermoFisher Scientific, Waltham, MA, USA).

*Molecular Identification* The seven-day-old cultures were utilized for DNA isolation. Genomic DNA extraction from the isolates was carried out following mechanical lysis in CTAB buffer, employing a modified protocol previously outlined by Gerrits van den Ende and Hoog 1999). Identification was primarily conducted through PCR amplification and Sanger sequencing of specific DNA regions/genes. The identification was done using internal transcribed spacers 1 and 2, which encompass the 5.8S rDNA region (White et al. 1990). These genes were PCR-amplified and Sanger sequenced utilizing the primer sets ITS1/ITS4 (White et al. 1990).

Fungal species were determined by comparing the obtained sequences with those of the most closely related strains and other relevant sequences stored in the non-redundant GenBank nucleotide database, utilizing the BLAST algorithm (Altschul et al. 1990). All DNA sequences pertaining to the isolated strains from this study have been deposited in the GenBank database under accession numbers PP348129–PP348165.

**Fig. 1:**
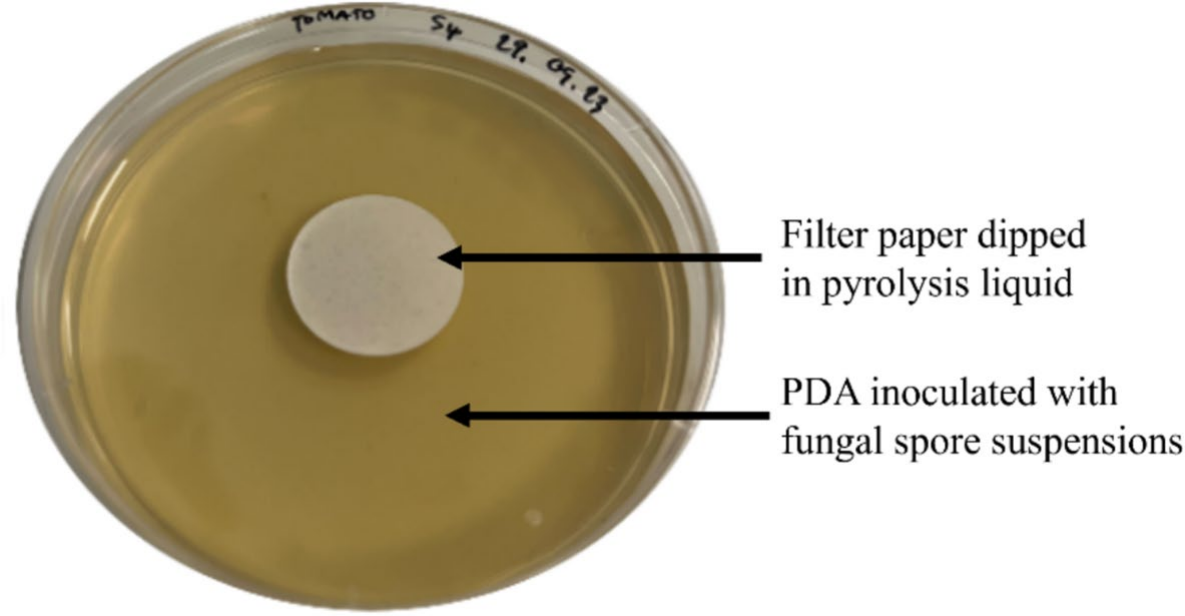
Illustration of the disk diffusion assay

*Disc diffusion test* Antifungal activity was assessed using the disc diffusion test adapted from the protocol described by Er et al. (2018). Briefly, 100 µL of the spore suspension of each culture isolated from bellpepper and fat, containing 10^8^ CFU/mL, was evenly spread over the surface of the PDA plate using a sterile glass spreader. Filter paper disks (1 cm in diameter) were dipped with the different pyrolysis liquid fractions and placed in the middle of Petri dishes on the agar surface as illustrated in Fig. 1. For each liquid fraction, five replicates were prepared per fungal strain. Reference samples containing fungal suspension and wet sterile filter paper (i.e., non-impregnated filter paper) were also prepared for comparison. The Petri dishes were then incubated in the dark at 25 °C in the growth chamber. After seven days of incubation, the presence of an inhibition zone around the filter paper disks was observed and evaluated.

Given that the pyrolysis liquid fractions collected in this study were highly diluted because of the high-water content of the samples, it was necessary to investigate the antifungal effect after partially evaporating the water to concentrate the active molecules potentially present in the samples. Based on preliminary results on the concentrations of fractions (< 2µm-particles), fraction 2 was identified as the most suitable for concentration. The initial volume was reduced by evaporating the water at 40 °C under nitrogen gas flow until the volume was reduced by fourfold, achieving an approximated concentration of 1% (v/v). The same disc diffusion test method was used to assess the antifungal potential of the concentrated sample against food spoilage fungi, specifically, *Cladosporium pseudocladosporioides and Penicillium* sp.

## Results and discussion

### Thermal degradation of bark material

**Fig. 2:**
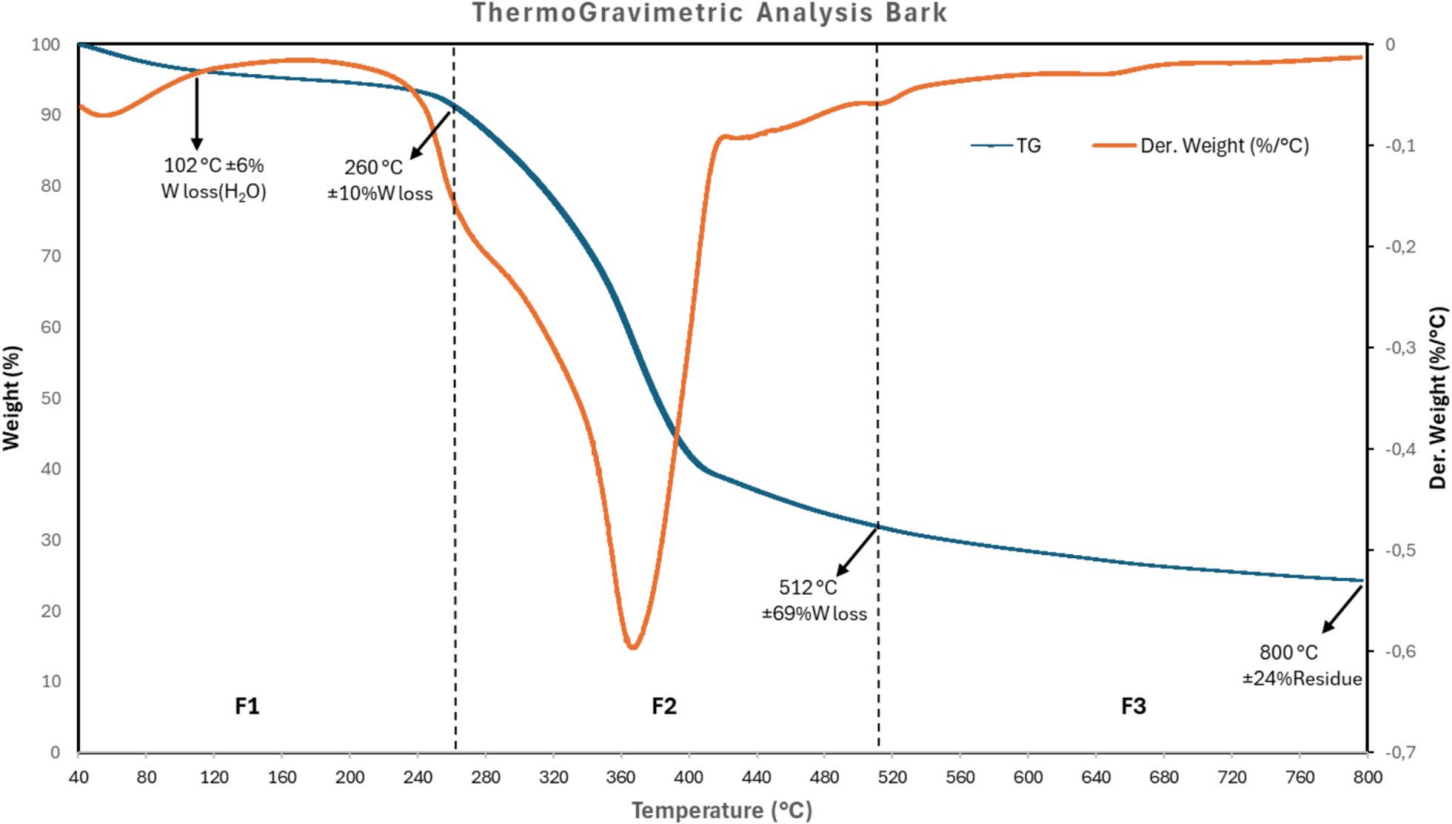
TG and dTG curves for raw bark

The evolution of thermal degradation behaviors of the raw bark material is represented by TG and dTG curves in Fig. 2.

The obtained degradation thermograms displayed a typical shape for lignocellulosic materials. Based on the dTG curve, the degradation of bark over a temperature range from 25 to 800 °C can be divided into four phases. The first phase, below 230 °C, was characterized by a slight endothermic peak. During this phase, the weight loss was about 6% and occurred mainly due to water evaporation and the release of light extractives present in the raw bark. Moreover, hemicelluloses eventually started to decompose during this phase given that they degrade at temperatures ranging from 220 to 315 °C (Yang et al. 2007). The second phase, from 230 to 300 °C, was characterized by a shoulder peak (Fig. 2). The weight loss was about 10% which was mainly caused by the degradation of remaining hemicelluloses. Indeed, it has been reported that hemicellulose degradation commonly starts with a shoulder peak with a maximum at 270 °C which corresponds to the destruction of glycosidic bonds between xylan units (Quan et al. 2016). In the third phase, from 300 to 420 °C, a sharp exothermic peak at a maximum temperature of 375 °C was observed in the dTG curve signifying a major weight loss (45%) caused by the release of volatiles bulk. Phase 3 represents the active phase being characterized by a fast degradation rate. The mass loss during phase 3 resulted from the decomposition of cellulose typically occurring between 315 °C and 400 °C (Yang et al. 2007). The degradation of lignin happened progressively during each of the three phases. Indeed, lignin usually decomposes slowly until 900 °C (Yang et al. 2007). Above 420 °C, in phase 4, the degradation rate slowed down to become almost constant by the end of the test (i.e., at 800 °C). This is called the passive phase and the weight loss was about 15%. The remaining solid residue at the end of the analysis at 800 °C was 23.5%. This residue consists of the char formed after thermal degradation of the biomass components. Prior research (Fetisova et al. 2023) reported that the residue from TGA analysis of fir, larch, spruce, and cedar bark were about 28, 33, 32, and 32%, respectively.

The TGA analysis showed the different thermal degradation phases of the bark. Different phases resulted from the decomposition of different components (i.e., hemicellulose, cellulose, and lignin). The same degradation trend occurs during the pyrolysis process in a tube furnace. Hence, pyrolysis liquid fractions collected at variable phases of the slow pyrolysis process are expected to exhibit differences in properties and molecular composition.

### Characteristics of the pyrolysis liquid

The pyrolysis liquid fractions have an intense smoky odor characteristic of charred materials, with a dark brown color for F2 and light-yellow color for F3 and F4. In contrast, F1 had a transparent appearance with no specific odor. Small residual particles were also present in all the fractions except F1 (Figure S1). These residues likely originated from the non- soluble tar fraction. Indeed, the pyrolysis liquid is composed of an aqueous phase (i.e., water-soluble compounds) and non-aqueous phase (i.e., non-soluble compounds with high molecular weight) (Demirbas 2007). During this study, the non-soluble tar part was not recovered because the amount was negligible in the collected aqueous fractions. Indeed, most of the tar remained in the pyrolysis system and condensation pipes. Therefore, fractions were filtered and only the soluble aqueous phase was subjected to characterization in this study. The characteristics of different pyrolysis liquid fractions are summarized in Table 2.

**Table 2:** Characteristics of the aqueous pyrolysis liquid fractions

	F1	F2	F3	F4
Temperature range, °C	25–260	260–512	512–800	800–25
Color	Transparent	Brown	Brown	Yellow
Mass concentration^1a^ (> 2µm-particles) mg/g	0.97 ± 0.05	6.82 ± 0.42	7.85 ± 0.45	3.12 ± 0.18
Mass concentration^1b^ (< 2µm-particles) mg/g	0.10 ± 0.01	2.40 ± 0.14	1.30 ± 0.08	0.50 ± 0.03
PH	6.37 ± 0.02	3.07 ± 0.03	3.23 ± 0.02	3.07 ± 0.01
Total phenolic compounds TPC [mg GAE/g extract]	n.d	6.46 ± 0.25	3.05 ± 0.14	0.77 ± 0.02

^1a^One-Way ANOVA: (F(3,8) = 297.98, p < 0.0001). Mass concentration shows statistically significant difference among the fractions. Tukey test revealed significant differences (p < 0.05) between all pairwise comparisons

^1b^One-Way ANOVA: (F(3,8) = 457.41, p < 0.0001). Mass concentration shows statistically significant difference among the fractions. Tukey test revealed significant differences (p < 0.05) between all pairwise comparisons

The extractables were filtered, and the concentrations of larger particles (> 2 µm), as well as the soluble fraction, were calculated. Notably, the higher concentration of large particles was observed in F3, indicating the presence of more insoluble material at that temperature range compared to F2. Conversely, the soluble fraction exhibited a higher concentration in F2, followed by F3, and the lowest in F1. The concentration results were in correlation with the degradation behaviors observed in the TGA analysis (Fig. 1). Indeed, F1 was collected at the first phase of the thermal conversion before the start of active degradation of bark components. Thus, negligible amounts of volatiles were generated which was reflected by low non-volatiles concentration. The highest weight loss was around 375 °C which was during the recovery of F2. The high weight loss correlated with the release of high amounts of volatiles which justifies the high non-volatiles concentration in F2. All samples had an acidic pH value except for F1 which was closer to neutrality (Table 2). The acidic pH likely indicates the presence of organic acids in the pyrolysis liquid fractions (F2, F3, and F4).

Regarding the TPC in each fraction, the values mirrored the pattern of soluble concentration. Substantial amounts were detected in F2 and F3, while only traces were found in F1 and F4. This suggests a correlation between the presence of phenolic compounds and the solubility characteristics of the fractions, with higher solubility fractions exhibiting greater TPC.

**Fig. 3:**
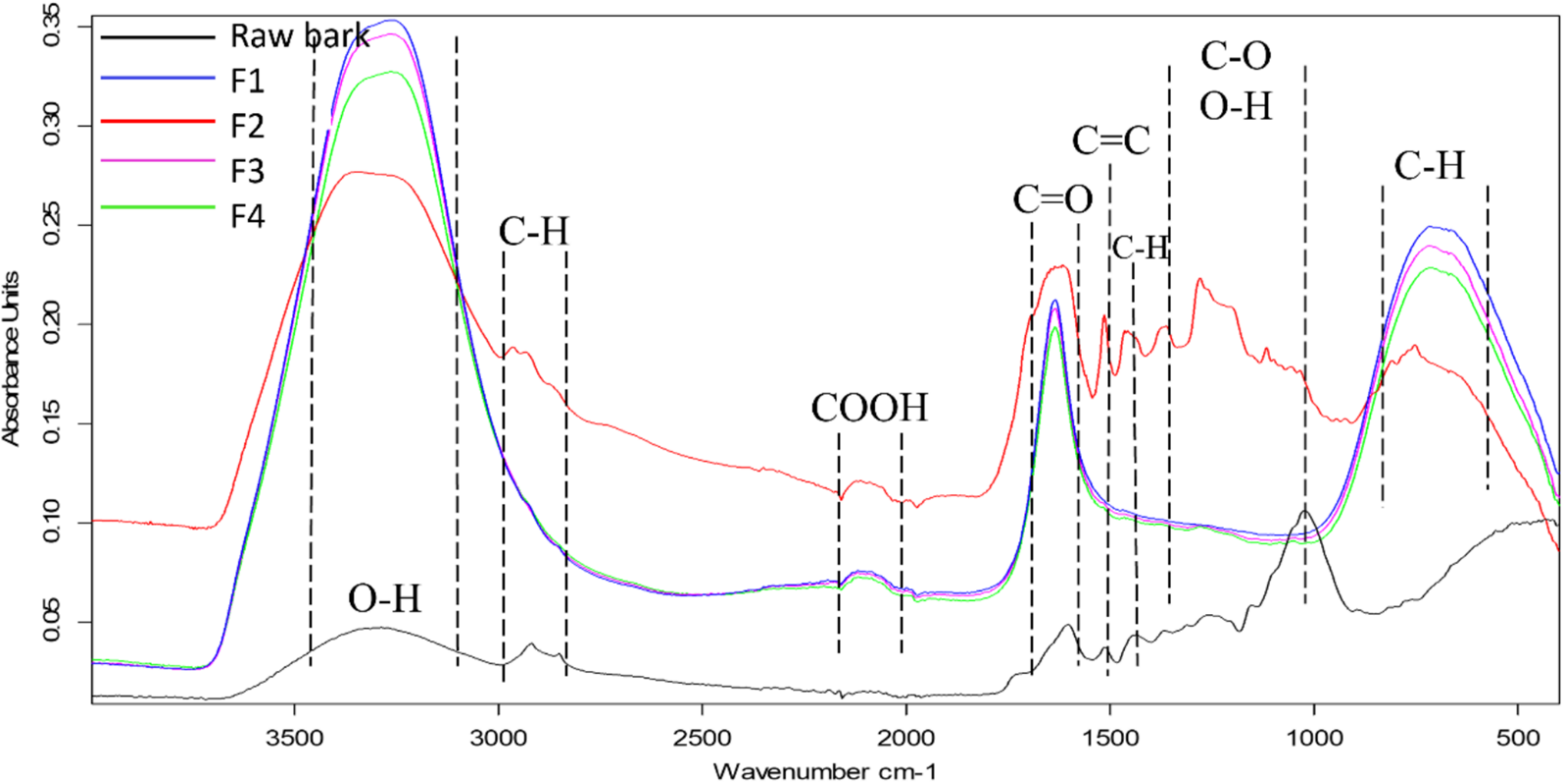
FTIR spectra of the raw bark material and the different pyrolysis liquid fractions

FTIR spectra of the raw bark material and the different pyrolysis liquid fractions are represented in Fig. 3.

A broad peak between 3400 cm^–1^ and 3200 cm^–1^ correspondent to O–H stretching vibration was observed in all samples and attributed to the high water content of the fractions. The O–H peak was less intense in the raw bark material due to its reduced moisture content compared to the liquid fractions. The peak between 2900 cm^–1^ and 2800 cm^–1^ was assigned to C-H stretching signaling the occurrence of alkanes. This peak was only detected in the raw bark material and in F2 fraction while it was absent in all other fractions. The band between 2050 cm^−1^ and 2200 cm^−1^ was absent in the original biomass, however, it was detected in all liquid samples. This peak was attributed to carboxyl groups. The occurrence of this band is associated with the presence of organic acids generated in volatiles during the thermal decomposition of cellulose and hemicellulose in the biomass. The presence of acidic compounds justifies the pH results (Table 2). Similarly, prior research (Stankovikj et al. 2017) reported the occurrence of carboxylic groups in the aqueous phase from hydrothermal treatment of wood materials. The peak between 1600 cm^−1^ and 1700 cm^−1^ was ascribed to C=O stretching vibrations correspondent to carbonyl groups from carboxylic acids, ketones, and aldehydes (Islam et al. 2010; Van Nam et al. 2020). This peak had low intensity in the raw bark while it was very intense in the pyrolysis liquid fractions. It can be associated with the degradation of hemicellulose and cellulose (Quan et al. 2016). The C=O stretching peak was sharp for all samples except for F2 where it was broader which likely indicates a higher variety of compounds containing C=O groups in F2 as compared to other fractions. The peak around 1500 cm ^−1^, attributed to C=C stretching of alkenes and aromatics (Dellarose Boer et al. 2021), was detected only in the raw material and F2 while it was absent in all other samples. Similarly, peaks at 1400 cm^−1^ associated with C-H groups from alkanes (Dellarose Boer et al. 2021; Islam et al. 2010) was only detected in the raw material and F2. In the original biomass, C=C and C–H groups can be attributed to hemicellulose and lignin (Özgenç Keleş et al. 2017). However, in F2, the peaks likely inform about compounds were likely released during hemicellulose and cellulose degradation which happens usually between 220 °C–315 °C and 315 °C–400 °C, respectively (Yang et al. 2007). Indeed, F2 was collected in the temperature range between 260 °C–512 °C. Peaks between 1000 cm^−1^ and 1350 cm^−1^ were attributed to C–O stretchings and O–H vibrations from alcohols (primary, secondary, and tertiary), esters, ether, and phenolic compounds (Dellarose Boer et al. 2021; Islam et al. 2010) and were detected only in F2. According to Quan et al. (2016), the band at 1259 cm^−1^ is related to the presence of phenols derived mainly from lignin degradation. At 1026 cm^−1^, an intense peak was observed in the raw bark spectrum and was attributed to the occurrence of C–O stretch from hemicellulose and cellulose (Özgenç Keleş et al. 2017). A broad peak between 600 cm^−1^ and 800 cm^−1^ was observed in all samples except for the raw bark and was ascribed to C-H groups of aromatic compounds.

FTIR spectra provided an overall evaluation about functional groups from compounds that could be present in the different pyrolysis liquid fractions. Overall, all samples displayed comparable spectra except for F2 which showed additional peaks suggesting the occurrence of higher concentrations of functional chemical groups as compared to other fractions.

### The chemical composition of the pyrolysis liquids

**Fig. 4:**
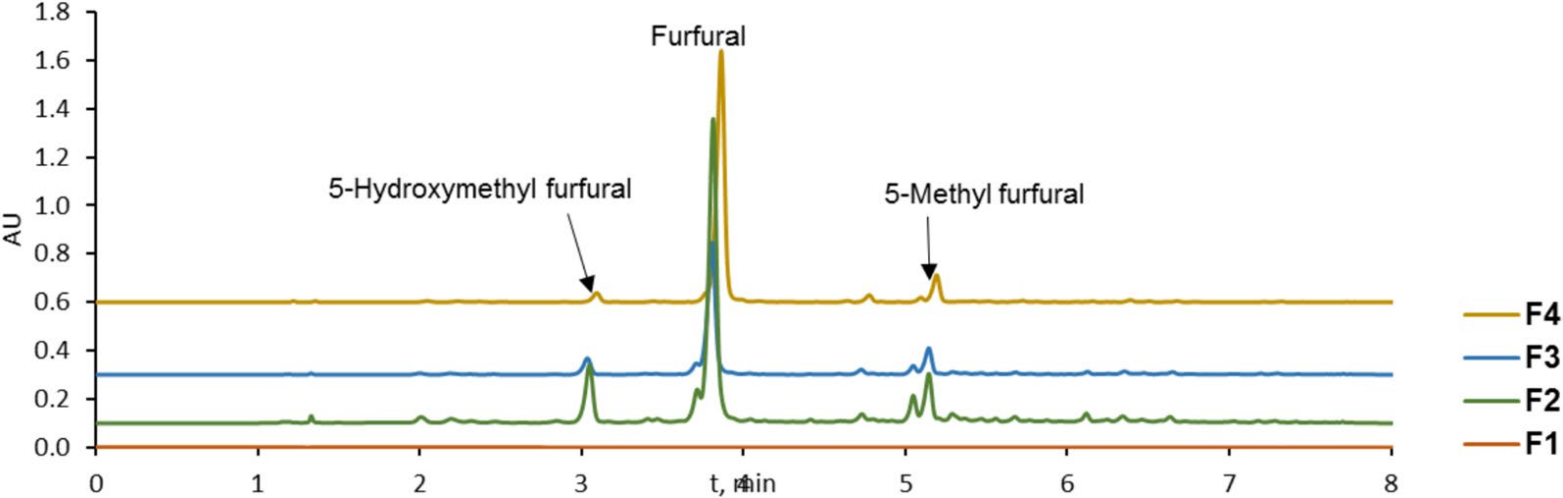
UHPLC-UV chromatograms of the pyrolysis liquid samples

The reversed phase UHPLC-UV clearly showed (Fig. 4) that the most abundant chemical compound in the samples is furfural (t_R_ 3.82 min), followed by 5-hydroxymethyl furfural (t_R_ 3.05 min), 5-methyl furfural (t_R_ 5.15 min) and an unknown compound with retention time (t_R_ 5.06 min) just before 5-methyl furfural. The mentioned chemicals were identified by their retention time and UV spectra match with the standards and were quantified based on the corresponding standard solutions. Table 3 summarizes the quantification results in all samples. First of all, the F1 sample did not contain detectable concentrations of furans or other compounds. In the other samples the concentration of summed furans was 240 µg/mL (F3), 412 µg/mL (F4) to 570 µg/mL (F2).

**Table 3:** Concentration of furans in the pyrolysis liquid samples

Sample	5-Hydroxymethyl furfural, µg/mL	Furfural, µg/mL	5-Methyl furfural, µg/mL
F1	n.d	n.d	n.d
F2	55 ± 4	460 ± 40	55 ± 5
F3	17 ± 1	190 ± 20	33 ± 2
F4	11 ± 1	370 ± 30	31 ± 1

Furfural is the main decomposition product of C5 sugars from hemicelluloses, but it can also arise from cellulose (Luo et al. 2019; Wang et al. 2024). Overall, the concentration of furfural was almost ten times higher than the concentration of other chemical compounds. Furfural, 5-HMF (5-hydroxymethylfurfural), and methyl furfural have been reported to exhibit significant antifungal activity by inhibiting fungal spore germination, hyphal elongation, and mycelial growth, making them key compounds of interest for bioactive applications (Jung et al. 2007; Oramahi et al. 2020). The ratio of 5-HMF and 5-MF changed during the carbonisation with 1:1 260–512 °C temperature range, followed by 1:2 at 512–800 °C temperature range and 1:3 during the final stage.

**Fig. 5:**
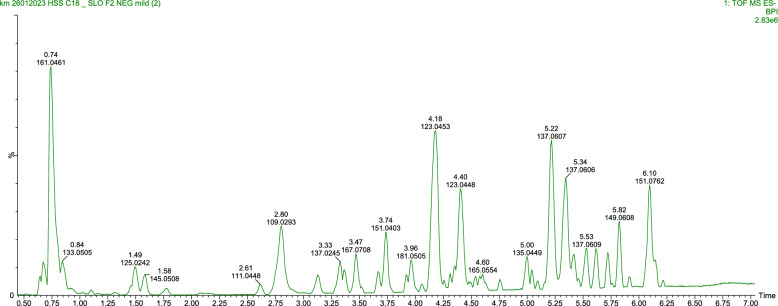
UHPLC-MS chromatogram of the pyrolysis liquid samples

The more sensitive mass spectrometry could additionally detect numerous chemicals (furans, and also phenols); however, their concentrations were very low (in general < 10 µg/mL). Figure 5 shows a UHPLC-MS chromatogram of the F2 sample with more than 30 resolved peaks. Significant qualitative differences of the samples were not observed. Since many of the compounds were isomers, conclusive identification was not possible, but the most likely chemical structures are summarised in Table 4. The UHPLC-MS analysis provides some insight into the chemical composition of the oxygenated phenols, which were quantified with the Folin–Ciocalteu method. The aromatic compounds included aldehydes and acids with differing number of methoxy- groups within structural isomers.

**Table 4:** Chemical Composition of F2 Sample and Relevance to Antifungal Activity

t_R_, min	Ion, Da	Formula [M-H]^−^	Possible ID	Relevance to Antifungal Activity
1.49	125.02	C_6_H_5_O_3_	Methyl furoic acid	Bactericidal, fungicidal and nematocidal agent (Fagbemi et al. 2022)
2.61	111.04	C_5_H_3_O_3_	Furoic acid	Antimicrobial agent (Chai et al. 2013)
2.80	109.03	C_6_H_5_O_2_	Acetylfuran	Antimicrobial agent (Alizadeh et al. 2020)
3.33	137.03	C_7_H_5_O_3_	Hydroxybenzoic acid	Strong antifungal properties by reducing the mycelial growth (Huo et al. 2023)
3.47	167.07	C_9_H_11_O_3_	Methylsyringol	Antimicrobial agent (Yang et al. 2016)
3.67/3.92	163.04	C_9_H_7_O_3_	Coumaric acid	Inhibite increase mycelial growth and spore germination (Hu et al. 2023)
3.74	151.04	C_8_H_7_O_3_	Hydroxyphenyl acetic acid	Could inhibit growth of fungal pathogens (Qu et al. 2017)
3.96	181.05	C_9_H_9_O_4_	Homovanillic acid	Antimicrobial agent (Fitzgerald et al. 2005)
4.18/4.40	123.04	C_7_H_7_O_2_	Guaiacol	Antimicrobial agent (Fitzgerald et al. 2005)
4.76	165.06	C_9_H_9_O_3_	Dimethoxybenzaldehyde	Antimicrobial agent (Qu et al. 2017)
5.22/5.34	137.06	C_7_H_5_O_3_	Dihydroxybenzaldehyde	Antimicrobial agent (Qu et al. 2017)
5.61/5.72	149.06	C_9_H_9_O_2_	Dimethyl benzoic acid	Antimicrobial agent (Qu et al. 2017)
6.10/6.21	151.08	C_9_H_11_O_2_	Dimethoxy methylbenzene	Antimicrobial agent (Fitzgerald et al. 2005)

These compounds, including aldehydes and acids with varying numbers of methoxy-groups within structural isomers, are known to exhibit a range of bioactivities, including antifungal properties. The identified compounds, such as furoic acid, hydroxybenzoic acid, and methyl syringol, are recognized for their ability to inhibit fungal growth with potential application in controlling fungal pathogens, particularly food-decaying fungi.

A summary of the main compounds identified, their retention times, molecular formulas, and relevance to antifungal activity is presented in Table 4 below. These compounds could contribute to the bioactivity of the fractions, through various mechanisms, such as cell wall disruption, inhibition of fungal growth and enzymatic interference.

### Antifungal activity

**Fig. 6:**
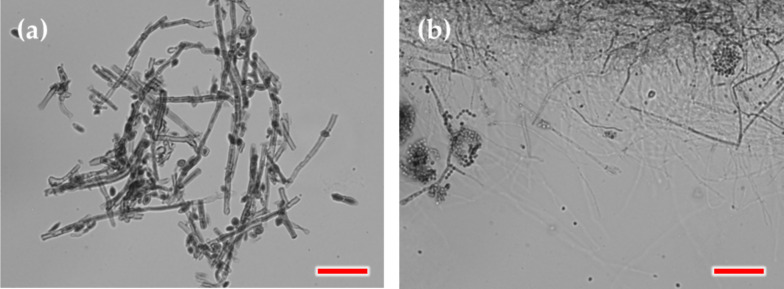
Microscopic images of the fungal species isolated from **a** bellpepper and **b** fat (Scale bar = 50 µm)

Based on morphological characteristics and DNA sequence analysis (Table S1), the fungal strains isolated from fat and bell pepper were identified as *Penicillium crustosum (P. crustosum)* and *Cladosporium* sp, respectively. The microscopic images of the different fungi after isolation and incubation are presented in Fig. 6.

**Fig. 7:**
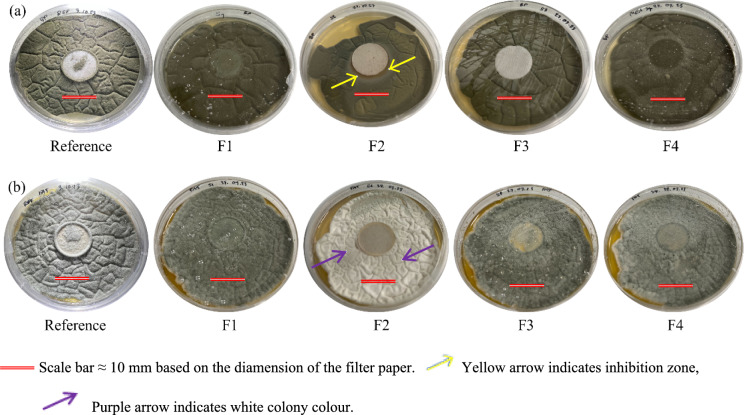
Growth of *Cladosporium* sp isolated from bell pepper (**a**) and *Penicillium crustosum* isolated from fat (**b**) on PDA with paper discs (reference) and paper discs impregnated with liquid fractions (F1–F4). Note: Growth after seven days of incubation in the dark under controlled climate conditions (25 °C and 90% RH) in the growth chamber. Each fraction and reference were tested in triplicated

Fungal growth in the presence of filter papers impregnated with pyrolysis liquid fractions was observed after seven days of incubation (Fig. 7). The antifungal activity of the liquid fractions was not considerable, however, fraction F2 showed a minor inhibitory effect on the growth of *Cladosporium* sp.*,* as evidenced by the small inhibitory zone of approximately 1 mm surrounding the 10 mm the filter paper (Fig. 7a). This effect was likely due to the high total phenolic compounds content (Tables 2, 4) and high concentration of furfural (Table 3), both known microbial inhibitors, in this fraction (Suresh et al. 2019). Additionally, despite no observable inhibition zone for *P. crustosum* in fraction F2, this fraction might have affected the morphology of the fungus as evidenced by the white colony colour (Fig. 7b). Notably, antifungal effects were not observed in the plates containing filter paper discs with fraction F1, F3, and F4. According to our observations, the antifungal activity of liquid by-products from bark carbonization was negligible which was likely due to the low concentration of active compounds given that the collected fractions were tested without further concentration or purification.

The lack of significant antifungal activity in F1, F3, and F4 can be attributed to a combination of factors, including the specific temperature ranges used for collection, the solubility of bioactive compounds, and their chemical stability. F1 primarily contains low-molecular-weight volatiles, such as organic acids and alcohols; however their low concentration and the relatively neutral pH of the fraction (6.37) limited their bioactivity. F3 and F4 were collected at higher temperature ranges, where lignin undergoes significant thermal degradation leading to the formation of high-molecular-weight polyaromatic compounds. F3 primarily consists of partially degraded lignin derivatives, including methoxyphenols and condensed phenolic structures, which are less water-soluble and more prone to thermal condensation or polymerization, thus reducing their antifungal efficacy. F4, collected at the highest temperature range, contains a greater proportion of highly polymerized and cross-linked aromatic compounds, reducing its solubility, diffusion and bioavailability, limiting its interaction with fungal cells.

**Fig. 8:**
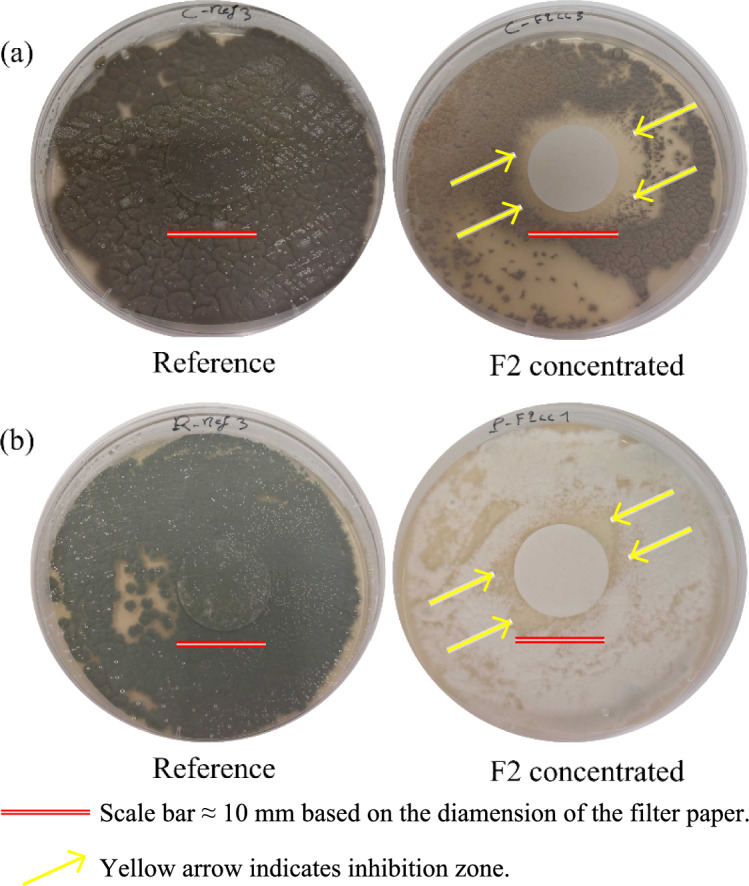
Growth of **a** *Cladosporium pseudocladosporioides* and **b** *Penicillium* sp. after seven days of incubation in the presence of concentrated F2 sample. Note: Growth in the dark under controlled climate conditions (25 °C and 90% RH) in a growth chamber. Each fraction and reference were tested in triplicated

In contrast, F2 corresponds to the decomposition of hemicellulose, cellulose, and partially lignin, capturing water-soluble phenolics and furans in their most bioactive forms. Among the fractions, F2 exhibited the highest abundance of bioactive compounds with known antifungal properties. This chemical profile, combined with its intermediate volatility and thermal stability, identified F2 as the most promising candidate for further testing. The investigation of concentrated F2 fraction against *Cladosporium pseudocladosporioides* and *Penicillium* sp showed more promising results (Fig. 8).

A total invasion of the surface was observed in the reference while an inhibition zone of approximately 2 mm was observed around the filter paper impregnated with the sample exposed to *C. pseudocladosporioides*. Further evidence of the effect of fraction F2 on the morphology of *Penicillium* was observed in the colony of *Penicillium* sp. treated with a concentrated solution of fraction F2 (Fig. 8b). The treated colony exhibited a white appearance whereas the control colony retained its characteristic green color, similar to that seen in *P. crustosum* exposed to fraction F2 (Fig. 7b). This phenotypic variation can be attributed to the phenotypic plasticity of *Penicillium*, a fundamental characteristic that allows fungi to modulate their morphology, physiology, and behavior in response to environmental stimuli (Slepecky and Starmer 2009).

Many studies have reported that phenolic compounds derived from the carbonization of wood exhibit significant antifungal activity. Suresh et al. (2019) demonstrated that acidic wood vinegar from softwood carbonization at 1% (v/v) concentration completely inhibited three different fungal strains (*Trametes versicolor, Aspergillus niger, and Aspergillus fumigatus*). It was suggested that the growth of fungi was limited by the antioxidative property of the phenolics which was favored by the acidic media (pH of 3.7). Similarly, Silva et al., (2021) found that castor seed cake carbonization liquids inhibited *Cryptococcus* species, showing that samples contained mainly N- and O-heterocycle compounds were successfully inhibiting their fungal growth. However, the fungal cultures in the reported study (Silva et al. 2021) were incubated for 24h only and further research is needed to confirm the long-term efficacy of such carbonization liquids. Dellarose Boer et al (2021) reported that sugarcane pyrolysis liquid (0.20%–0.25% v/v) effectively inhibited the growth of *Coniophora puteana* and *Trametes versicolor* in wood protection applications.

Derbali et al. (2024) revealed that antifungal properties against phytopathogenic fungi of the carbonization extract obtained from *Pinus halepensis* and *Acacia cyanophylla* are attributed to their high content of phenolic compounds. Kim et al. (2012) mentioned that phenolic compounds in bio-oil produced from the tulip tree (*Liriodendron tulipifera*) play an imortant role in protecting against brown rot (*Tyromyces palustris*) and white rot (*Trametes versicolor*) in soil block tests. Okutucu et al. (2011) demonstrated that the antifungal activity of bio-oil obtained from the pyrolysis of pistachio shells can be attributed to its high phenolic content. This bio-oil exhibited significant antifungal effects against a range of fungi, including the saprophytic fungus (*Aspergillus niger*), the phytopathogenic fungus (*Trichoderma viridae*), the white rot fungus (*Coriolus versicolor*), and the dermatophytic fungus (*Trichophyton rubrum*).

While these studies reported successful results, other studies found non encouraging results. For instance, wood vinegar was not effective in inhibiting *Aspergillus flavus*, *Aspergillus parasiticus*, and aflatoxin contamination in peanut plants when used in soil (Jothityangkoon et al. 2008). Likewise, Hou et al. (Hou et al. 2018) found that the antimicrobial activity of wood vinegar collected at different pyrolysis temperature was more promising in the case of bacteria compared to fungi (*Penicillium*, *Aspergillus*, and *Rhizopus*). The highest inhibitory effect was obtained for a sample collected at 450–480 °C with an inhibition diameter of 12.5 mm. In comparison, the findings of this study confirm that concentration of pyrolysis liquids significantly influences antifungal efficacy. While F2 alone exhibited minimal inhibition, its concentrated form (1% v/v) resulted in measurable inhibition zones (~ 2 mm), aligning with previous research on carbonization liquids.

Although this study focused on the lab-scale fractionation and characterization of the liquid by-products from the pyrolysis process, the scalability of the process for industrial applications deserves consideration. The pyrolysis process itself is inherently scalable, as demonstrated by its widespread adoption in the production of biochar, bio-oils, and other bio-based products. However, the consistent collection of liquid fractions with controlled chemical compositions requires precise process control, particularly in maintaining stable temperature ranges and gas flow rates during the pyrolysis process.

In industrial conditions, the implementation of automated fraction collection systems and real-time monitoring of chemical profiles (e.g. through in-line Infrared or gas analysis) could enhance reproducibility and ensure consistent quality of the fractions. Moreover, additional studies will be needed to optimize the operational parameters, such as heating rates, feedstock moisture content, and reactor configurations, to ensure consistent yields of bioactive compounds. In addition, further purification and rigorous toxicity testing are needed to meet food safety standards before scaling up for practical applications.

## Conclusion

In this study aqueous pyrolysis liquid from softwood bark was characterized and investigated for its antifungal capacity against food decaying fungi. The liquid fraction F2 collected in the temperature range of 260–512 °C had the highest total phenolic content among other fractions (6.46 mg GAE/g extract). Results were associated with the high degradation rate of the bark components (hemicellulose and cellulose) in the temperature range where F2 was obtained. F2 sample also contained the highest amount of total furans (570 µg/mL), with furfural, 5-hydroxymethyl furfural, and 5-methyl furfural identified ad the main compounds. The inhibitory effect of F2 against *Penicillium crustosum* and *Cladosporium* sp isolated from animal fat and bell pepper, respectively was minor. However, when F2 was further concentrated, an inhibition effect on fungal growth was observed. Overall, the pyrolysis liquid demonstrated promising antifungal activity with potential applications against food-decaying fungi. However, advanced post-treatment processes, such as filtration, purification, toxicity analysis, are required to comply with food safety standards and enable its use as a food preservative. Furthermore, additional application tests are needed to validate the antifungal efficacy of these fractions under practical conditions and evaluate their scalability for industrial or agricultural applications. Future research should also explore broader applications beyond food preservation, such as wood preservation and agricultural fungicides, as well as their potential use in sustainable packaging coatings or post-harvest treatments to extend the shelf life of fresh produce. These applications could enhance the industrial relevance of pyrolysis liquid while contributing to the development of bio-based antimicrobial solutions.

## Supplementary Information

Supplementary material 1.

## Data Availability

Not applicable.
