# Supplementary material for: Properties and anti-fungal activity of liquid by-products from softwood bark carbonization
Source: Bioresour Bioprocess. 2025 Apr 24;12(1):39. doi: 10.1186/s40643-025-00875-8 (PMC12021764; doi:10.1186/s40643-025-00875-8)
Supplement: Supplementary file 1 — Supplementary material 1. [file 40643_2025_875_MOESM1_ESM.docx]

**Properties and anti-fungal activity of liquid by-products from softwood bark carbonization**


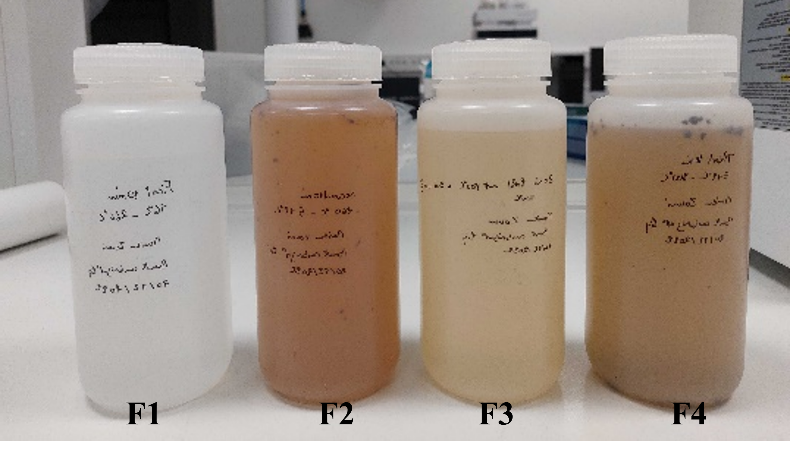


**Figure S1.** Liquid fractions obtained from pyrolysis of bark material.

**Table S1.** DNA sequences of the isolated fungi.

| **Isolate name** | **Genus** | **Species** | **ITS** |
| --- | --- | --- | --- |
| F41 | Cladosporium | sp. | AGGTCACCTTAGAAATGGGTTTGTTTTACGGCGTAGCCTCCCGAGCACCCTTTAGCGAATAGTTTCCACAACGCTTAGGGGACAGAAGACCCAGCCGGTCGATTTGAGGCACGCGGCGGACCGCGTTGCCCAATACCAAGCGAGGCTTGAGTGGTGAAATGACGCTCGAACAGGCATGCCCCCCGGAATACCAGGGGGCGCAATGTGCGTTCAAAGATTCGATGATTCACTGAATTCTGCAATTCACATTACTTATCGCATTTCGCTGCGTTCTTCATCGATGCCAGAACCAAGAGATCCGTTGTTAAAAGTTTTAATTTATTAATTAAGTTTACTCAGACTGCAAAGTTACGCAAGAGTTTGAAGTGTCCACCCGGAGCCCCCGCCCGAAGGCAGGGTCGCCCCGGAGGCAACAGAGTCGGACAACAAAGGGTTATGAACATCCCGGTGGTTAGACCGGGGTCACTTGTAATGATCCCTCCGCAGGTTCACCTACGGAGACCTTGTTACRACT |
| F33 | Penicillium | crustosum (sp.) | GTCACCTGGATAAAAATTTGGGTTGATCGGCAAGCGCCGGCCGGGCCTACAGAGCGGGTGACAAAGCCCCATACGCTCGAGGACCGGACGCGGTGCCGCCGCTGCCTTTCGGGCCCGTCCCCCGGAGATCGGGGGACGGGGCCCAACACACAAGCCGGGCTTGAGGGCAGCAATGACGCTCGGACAGGCATGCCCCCCGGAATACCAGGGGGCGCAATGTGCGTTCAAAGACTCGATGATTCACTGAATTTGCAATTCACATTACGTATCGCATTTCGCTGCGTTCTTCATCGATGCCGGAACCAAGAGATCCGTTGTTGAAAGTTTTAAATAATTTATATTTTCACTCAGACTTCAATCTTCAGACAGAGTTCGAGGGTGTCTTCGGCGGGCGCGGGCCCGGGGGCGTAAGCCCCCCGGCGGCCAGTTAAGGCGGGCCCGCCGAAGCAACAAGGTAAAATAAACACGGGTGGGAGGTTGGACCCAGAGGGCCCTCACTCGGTAATGATCCTTCCGCAGGTTCACCTACGGAAACCTTGTTACGACT |
